# Calcification may not protect the aorta

**DOI:** 10.1093/ehjcr/ytae391

**Published:** 2024-08-01

**Authors:** Long Cao, Xiao Bi, Ruimin Wang, Wei Guo

**Affiliations:** Department of Vascular and Endovascular Surgery, Chinese PLA General Hospital, Fuxing Road 28, Beijing 100853, China; Department of General Surgery, The 983rd Hospital of Joint Logistic Support Force of PLA, Tianjin 300142, China; Department of Nuclear Medicine, The First Medical Centre, Chinese PLA General Hospital, Beijing, China; Department of Nuclear Medicine, The First Medical Centre, Chinese PLA General Hospital, Beijing, China; Department of Vascular and Endovascular Surgery, Chinese PLA General Hospital, Fuxing Road 28, Beijing 100853, China

A 77-year-old woman who had been diagnosed with aortic intramural haematoma a year earlier was admitted with acute type B aortic dissection of unknown cause. Computed tomography (CT) angiography of the acute aortic intramural haematoma (*Figure 1A–E*; Supplementary material online, *Video S1*) had shown a heavily calcified aorta with no obvious intimal tear and complete thrombosis of a false lumen. Computed tomography angiography performed soon after onset of acute aortic dissection (*Figure 1F–J*; Supplementary material online, *Video S2*) showed localized dissection with an obvious intimal tear and significant progression of calcification in the descending thoracic aorta (*Figure 1B* and *C* vs. *Figure 1F–I*). Comparison of the two sets of CT angiographic images showed that the intimal tear started close to the border of an area of increased macrocalcification (*Figure 1D* and *E* vs. *Figure 1G–H*). ^18^F-sodium fluoride positron emission tomography/CT scans obtained before endovascular therapy revealed numerous invisible microcalcifications adjacent to the intimal tear and surrounding the macrocalcification area (*Figure 1K–M*), indicating an increased calcification volume.^1^ Interestingly, the standardized uptake value for ^18^F-sodium fluoride was highest (maximum, 2.85) at the edge of the intimal tear on the true lumen side (*Figure 1M*, white arrow), indicating that this site was vulnerable to dissection.^2^

**Figure 1:**
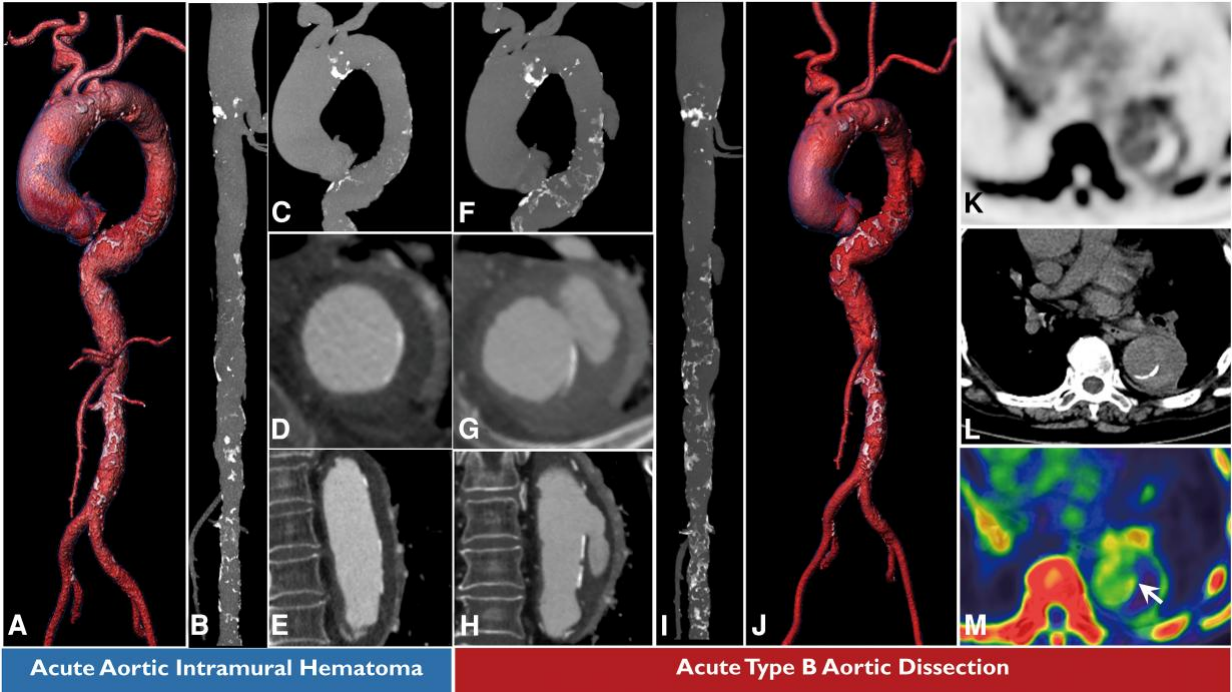
(*A*) Three-dimensional volume rendering of computed tomography angiography of the previously acute aortic intramural haematoma. (*B–C*) Three-dimensional volume rendering of the stretched aorta and thoracic aorta. (*D*) Reconstructed axial image (perpendicular to the aortic centre lumen line) prior to the intimal tear formation. (*E*) Sagittal image of the aortic intramural haematoma. (*F*) Three-dimensional volume rendering of computed tomography angiography of the thoracic aorta after dissection. (*G*) Reconstructed axial image (perpendicular to the aortic centre lumen line) to show the intimal tear. (*H*) Sagittal image of the post-dissected aorta. (*I–J*) Three-dimensional volume rendering of computed tomography angiography of acute aortic dissection. (*K*) ^18^F-sodium fluoride uptake at the site of intimal tear on positron emission tomography image. (*L*) Axial image at the site of intimal tear on computed tomography image. (*M*) Pseudocolour fusion of positron emission tomography and computed tomography images at the same axial plane of the intimal tear. PET, positron emission tomography; CT, computed tomography.

Evidence regarding the role of aortic calcification in development of acute aortic syndrome is limited. Rather than serving as ‘protective armour’, increased calcification in the thoracic aorta may increase the risk of acute aortic syndrome or aggravate pre-existing aortic disease. This risk may be determined by the underlying activity of invisible microcalcification.

## Supplementary Material

ytae391_Supplementary_Data

## Data Availability

The original data are available by the authors upon reasonable request.
